# Increased persistence of avoidance behaviour and social deficits with *L.rhamnosus* JB-1 or selective serotonin reuptake inhibitor treatment following social defeat

**DOI:** 10.1038/s41598-020-69968-y

**Published:** 2020-08-10

**Authors:** Yunpeng Liu, Kailey Steinhausen, Aadil Bharwani, M. Firoz Mian, Karen-Anne McVey Neufeld, Paul Forsythe

**Affiliations:** 1 McMaster Brain-Body Institute, The Research Institute of St. Joseph’s Hamilton, Hamilton, Canada; 2 Department of Pathology and Molecular Medicine, McMaster University, Hamilton, Canada; 3 Michael G. DeGroote School of Medicine, McMaster University, Hamilton, Canada; 4 Department of Medicine, McMaster University, Hamilton, Canada; 5 Firestone Institute for Respiratory Health, St. Joseph’s Healthcare and Department of Medicine, McMaster University, Hamilton, Canada

**Keywords:** Psychiatric disorders, Post-traumatic stress disorder, Social behaviour

## Abstract

Chronic social defeat (CSD) in mice has been suggested as a model for studying post-traumatic stress disorder (PTSD). Our previous work indicated that exposure to *Lactobacillus rhamnosus* JB-1 (JB-1) during CSD can attenuate subsequent behavioural and immune disruption, suggesting a potential for microbe based therapeutic approaches in PTSD. In the current study, we assessed the ability of JB-1 to mitigate the behavioral consequences of CSD when treatment is instigated in the early post-stress period and compared the probiotic effects with those of the selective serotonin reuptake inhibitor (SSRI), sertraline. JB-1 or sertraline were administered orally 48 h following 10-days of CSD in male C57BL/6 mice. Contrary to our hypothesis of a beneficial effect, 30 days of treatment with either JB-1 or sertraline increased the persistence of both aggressor avoidance and reduced sociability in defeated mice. This was accompanied by lower hippocampal mRNA expression for genes related to fear memory. Defeated mice treated with either JB-1 or sertraline also exhibited systemic immune changes, with a decrease in Th1 cells, activated monocytes, and the monocyte chemoattractant CCL2. This study identifies potentially detrimental effects of both JB-1 and sertraline if administered in the early post-trauma period and suggests caution should be applied when considering psychobiotic or SSRI based approaches for early intervention in trauma related psychiatric disorders.

## Introduction

Post-traumatic stress disorder (PTSD) is a debilitating condition that can develop following experience of, or exposure to, severely traumatic or life-threatening events. The disorder is characterized by clusters of symptoms including involuntary and intrusive memories of the trauma, avoidance of trauma-related stimuli and social detachment^1^. In addition, PTSD is often comorbid with anxiety and depression, and those with the disorder are up to six times more likely to commit suicide^2–4^.

Current treatment for PTSD includes cognitive behavioral therapy (CBT) and pharmacological interventions, with selective serotonin reuptake inhibitors (SSRIs) being the first-line in pharmacotherapy^5^. However, CBT has a relatively high drop-out rate in PTSD patients, especially in the population of veterans^6,7^, while the efficacy of SSRIs is also less than optimal, with only approximately half of patients responding to treatment and full remission achieved in 20–30%^8,9^. There is a clear need for more effective strategies to treat PTSD, or to prevent onset of the disorder following exposure to trauma.

The microbiota-gut-brain axis is a term used to describe a broad set of interactions between the gut microbiota and the central nervous system which involve endocrine, immune, and neural signalling pathways^10^. It is now established that exposure to psychological stressors can alter the composition of the gut microbiota while, conversely, alteration of the microbiota or exposure of the gut to specific bacteria can modulate brain chemistry, stress responses and anxiety/depressive-like behaviors^11^. Furthermore, probiotic and prebiotic treatments have been demonstrated to enhance stress resilience and mitigate PTSD relevant behaviors in animal models^12–15^. Such observations, together with evidence of altered microbiota profiles in subjects with psychiatric conditions including major depressive disorder (MDD) and PTSD, has prompted the exploration of microbe-based approaches to mental health^16^.

Oral treatment with *Lactobacillus rhamnosus* JB-1 (JB-1) has been demonstrated to lead to changes in neurotransmitters in the brains of mice and to have anxiolytic and antidepressant-like activity^17,18^. We previously demonstrated that feeding JB-1 prior to stress exposure could attenuate behavioural deficits and systemic immune alterations induced by CSD, an animal model with features of PTSD^15^. This previous study suggested a potential prophylactic role for beneficial bacteria in mitigating the detrimental effects of subsequent stress exposure in mice. Also, in the same study, we found that 28-day administration of JB-1 did not lead to significant differences in behaviors including sociability and aggressor-approach avoidance in mice in the absence of social defeat. However, the ability of the probiotic treatment to influence brain and behaviour when given following social defeat, and thus its potential as a treatment or early post-exposure intervention for stress and trauma related disorders, has not been assessed.

In the current study, we investigated the effects of post-defeat treatment with JB-1 on behavior, molecular alterations in the hippocampus, and immune changes in the mouse CSD model. We compared the effects of JB-1 to those of sertraline, an SSRI that is FDA approved for use in treatment of PTSD.

## Results

### JB-1 and sertraline treatment both increase persistence of aggressor avoidance and reduced sociability in mice when administered following chronic social defeat

**Figure 1:**
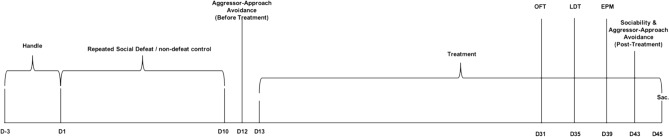
Timeline of the experimental protocol (*OFT* open field test, *LDT* light–dark test, *EPM* elevated plus maze).

The timeline of the experiments is illustrated in Fig. 1.

**Figure 2:**
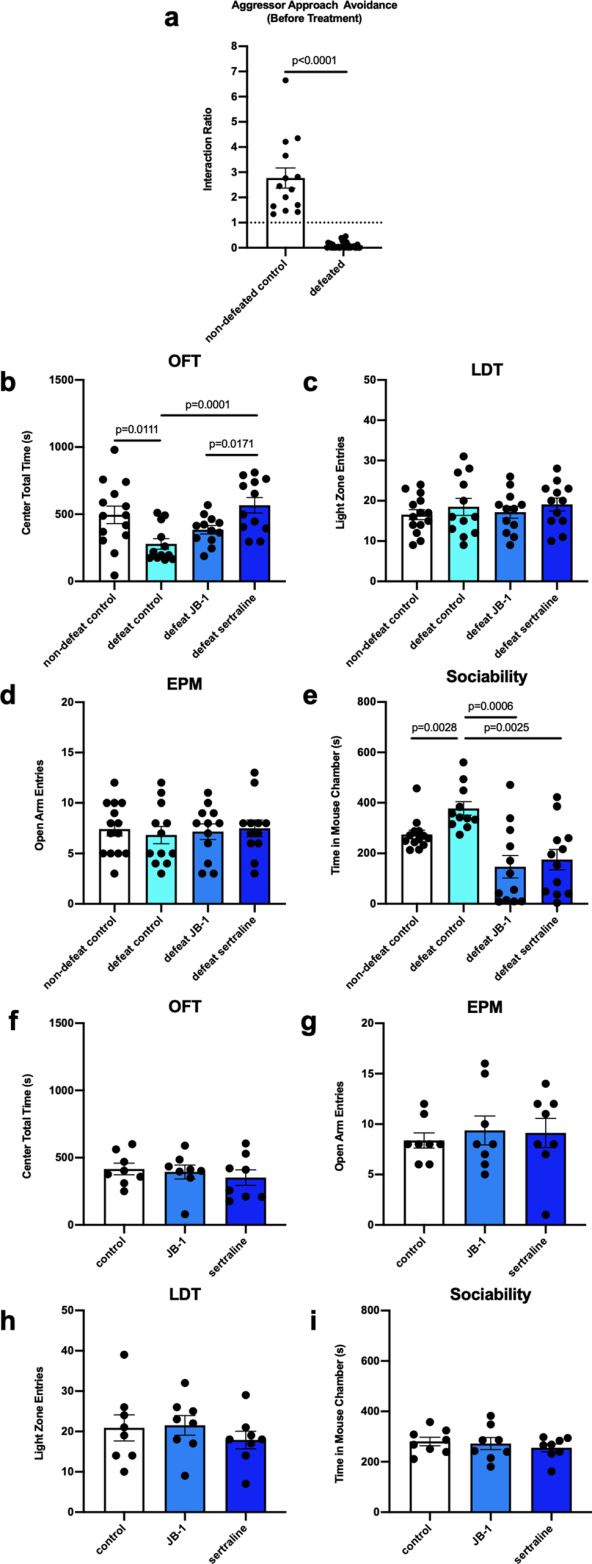
Anxiety-like and social behavior in CSD (**a**–**e**) and non-defeated mice (**f–i**) following treatment with JB-1 or sertraline. (**a**) Interaction ratio (time spent in aggressor area/time spent in empty area) of aggressor approach avoidance test, before treatment (n = 14 in non-defeat control group, n = 36 in defeated group). (**b**) Time spent in center area of open field test. (n = 14 in non-defeat control group, n = 12 in each of other groups). (**c**) Entries into light zone of light–dark test (n = 14 in non-defeat control group, n = 12 in each of other groups). (**d**) Entries into open arm of elevated plus maze (n = 14 in non-defeat control group, n = 12 in each of other groups). (**e**) Time spent in mouse chamber of 3-chamber sociability test (n = 13 in non-defeat control group, n = 12 in each of other groups). (**f**) Time spent in center area of open field test (n = 8). (**g**) Entries into light zone of light–dark test (n = 8). (**h**) Entries into open arm of elevated plus maze (n = 8). (**i**) Time spent in mouse chamber of 3-chamber sociability test (n = 8).

Animals were initially divided into 2 groups of non-defeated (n = 12) and defeated (n = 36) mice. Following CSD, an aggressor avoidance test was used to confirm that mice were susceptible to social defeat prior to inclusion in post-defeat treatment groups, with an interaction ratio of < 1 considered as avoidance behaviour^19^. 48 h following the final defeat session, defeated mice displayed avoidance behavior which was significantly different from non-defeat control (non-defeat control: 2.77 ± 0.40, max: 6.65, minimum: 1.34, defeated: 0.10 ± 0.02, max: 0.45, minimum: 0.00, g = 3.40, *p* < 0.0001) (Fig. 2a). Having confirmed avoidance behaviour in all defeated mice, these animals were then randomly assigned to the 3 different treatment groups (control, JB-1 or sertraline treated).

Following 18 days of treatment (day 21 post-defeat), mice underwent behavior tests. In the OFT the defeated control group spent significantly less time in the center zone compared to the non-defeated group (non-defeat control: 496.08 ± 64.92 s, defeat control: 278.81 ± 39.00 s, g = 1.08, *p* = 0.0111). Treatment with JB-1 had no significant effect on the behaviour of defeated mice in the OFT, however sertraline increased the time defeated mice spent in the center zone as compared to defeat control mice (defeat sertraline: 566.87 ± 56.96 s, F (2, 33) = 4.294, η^2^*p* = 0.21, g = 1.70, *p* = 0.0001), indicative of an anxiolytic effect of the SSRI (Fig. 2b). On post-defeat days 24 and 28 we conducted the LDT and EPM respectively. There was no significant difference between the behavior of defeated and non-defeated mice in either test and no effect of treatment with either JB-1 or sertraline (Fig. 2c, d).

**Figure 3:**
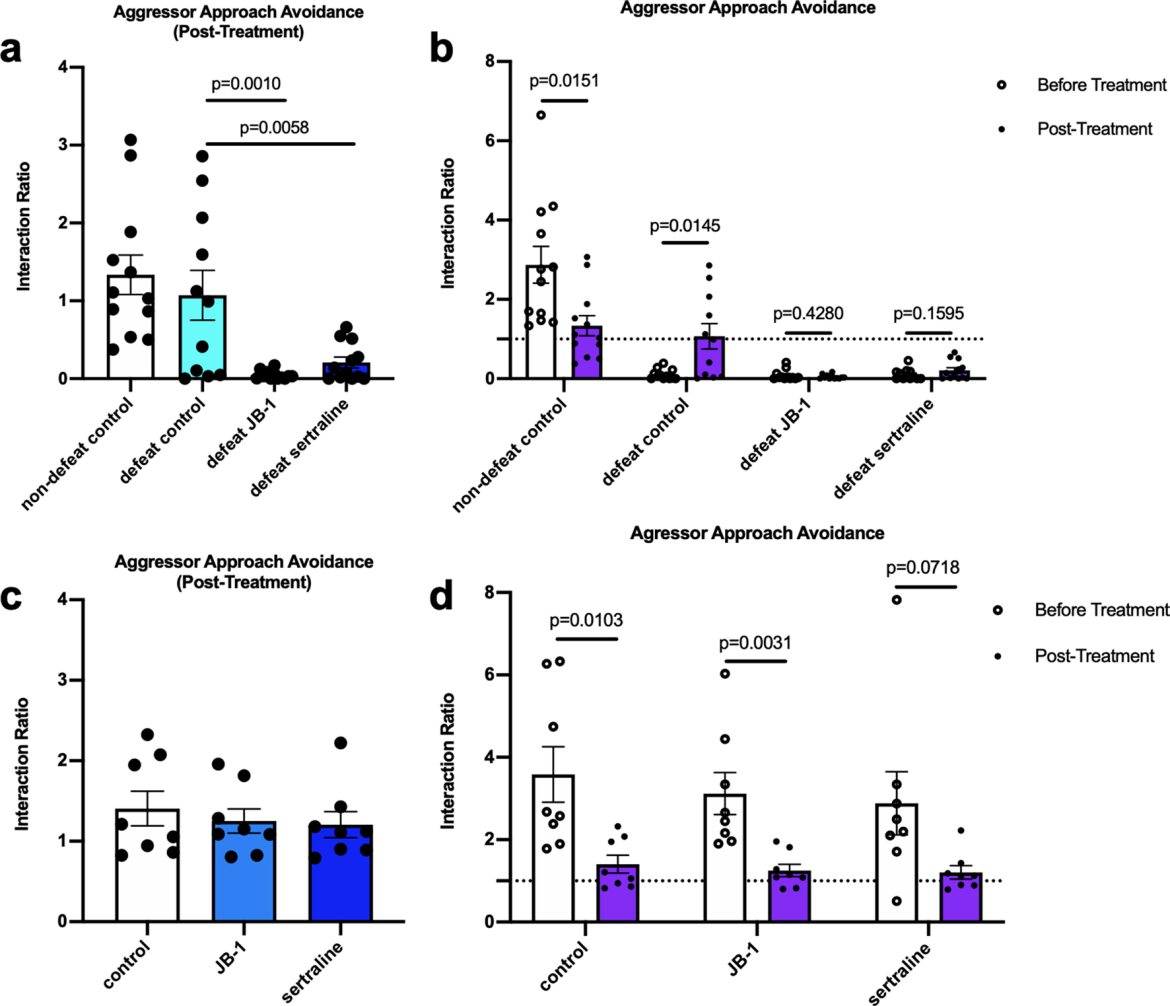
Post-treatment interaction ratio in the aggressor avoidance test and comparison of interaction ratio, pre and post-treatment by paired *t* test, in CSD (**a**,**b**) exposed mice (n = 11–12), and non-defeated (**c**,**d**) cohorts (n = 8).

After 30 days of treatment (post-defeat day 32), all mice were tested in the 3-chamber sociability test and retested for aggressor-approach avoidance behaviour. The sociability test demonstrated a significant increase in social behaviour in defeated mice as compared to non-defeat control (non-defeat control: 274.76 ± 17.33 s, defeat control: 378.01 ± 26.43 s; g = 1.38, *p* = 0.0028) at post-defeat day 32. This finding is in contrast to a cohort of mice tested for social behavior 1 day after social defeat, where a significant impairment in social interaction was observed (see Supplementary Fig. S1). Compared to the defeat control group at 32 days post-defeat, mice treated with either JB-1 or sertraline spent significantly less time in the mouse chamber (F (2, 32) = 1.765, η^2^*p* = 0.10, JB-1: 146.94 ± 44.83 s, g = 1.81, *p* = 0.0006; sertraline: 175.42 ± 40.10 s, g = 1.73, *p* = 0.0025) (Fig. 2e). In the second aggressor approach avoidance test there was no longer a significant difference in the mean interaction ratio between the defeat and non-defeat controls (non-defeat control: 1.34 ± 0.25, max: 3.07, min: 0.37; defeat control: 1.07 ± 0.32, max: 2.86, min: 0.00; g = 0.27, *p* = 0.5216), however the difference was maintained in both the JB-1 and sertraline treated mice, with both these groups also demonstrating significantly lower interaction ratios than defeat control mice. (F (2, 32) = 21.44, η^2^*p* = 0.57, JB-1: 0.047 ± 0.016, g = 1.40, *p* = 0.0010; sertraline: 0.21 ± 0.070, g = 1.12, *p* = 0.0058) (Fig. 3a). Comparison of the time spent in interaction zone between tests before and post-treatment by paired *t* test showed a significant decrease in non-defeat control group, but a significant increase in defeat control group, which was not shown in defeat JB-1 or sertraline group (non-defeat control: g = 1.19, *p* = 0.0151; defeat control: g = 1.28, *p* = 0.0145; defeat JB-1: g = 0.36, *p* = 0.04280; defeat sertraline: g = 0.55, *p* = 1535) (Fig. 3b). Moreover, 32 days after defeat, 5 of 11 (45.4%) of mice in defeat control group showed an interaction ratio > 1, which was not observed in mice treated with either JB-1 or sertraline (see Supplementary Fig. S2a). The locomotor ability was not changed among groups according to basic movements of OFT (Supplementary Fig. S2b).

To determine if the effects observed in JB-1 and sertraline groups occurred independently of CSD we examined additional cohorts of mice, housed identically to the CSD mice but without exposure to aggressors. In these non-defeated mice, we did not find any significant difference in behaviour following treatment with JB-1 or sertraline. Figures 2f–i and 3c. As with our initial non-defeated cohorts, the interaction ratio of aggressor avoidance was reduced between the first and second test (control: before treatment: 3.583 ± 0.674, post-treatment: 1.406 ± 0.215, g = 1.54, *p* = 0.0131; JB-1: before treatment: 3.119 ± 0.512, post-treatment: 1.252 ± 0.150, g = 1.75, *p* = 0.0031), while a trend of reduction in sertraline group (before treatment: 2.883 ± 0.767, post-treatment: 1.206 ± 0.162, g = 1.07, *p* = 0.0718) (Fig. 3d). Also, the locomotor ability was not affected by either JB-1 or sertraline (Supplementary Fig. S2c).

### JB-1 and sertraline alter hippocampal gene expression in mice exposed to social defeat

**Figure 4:**
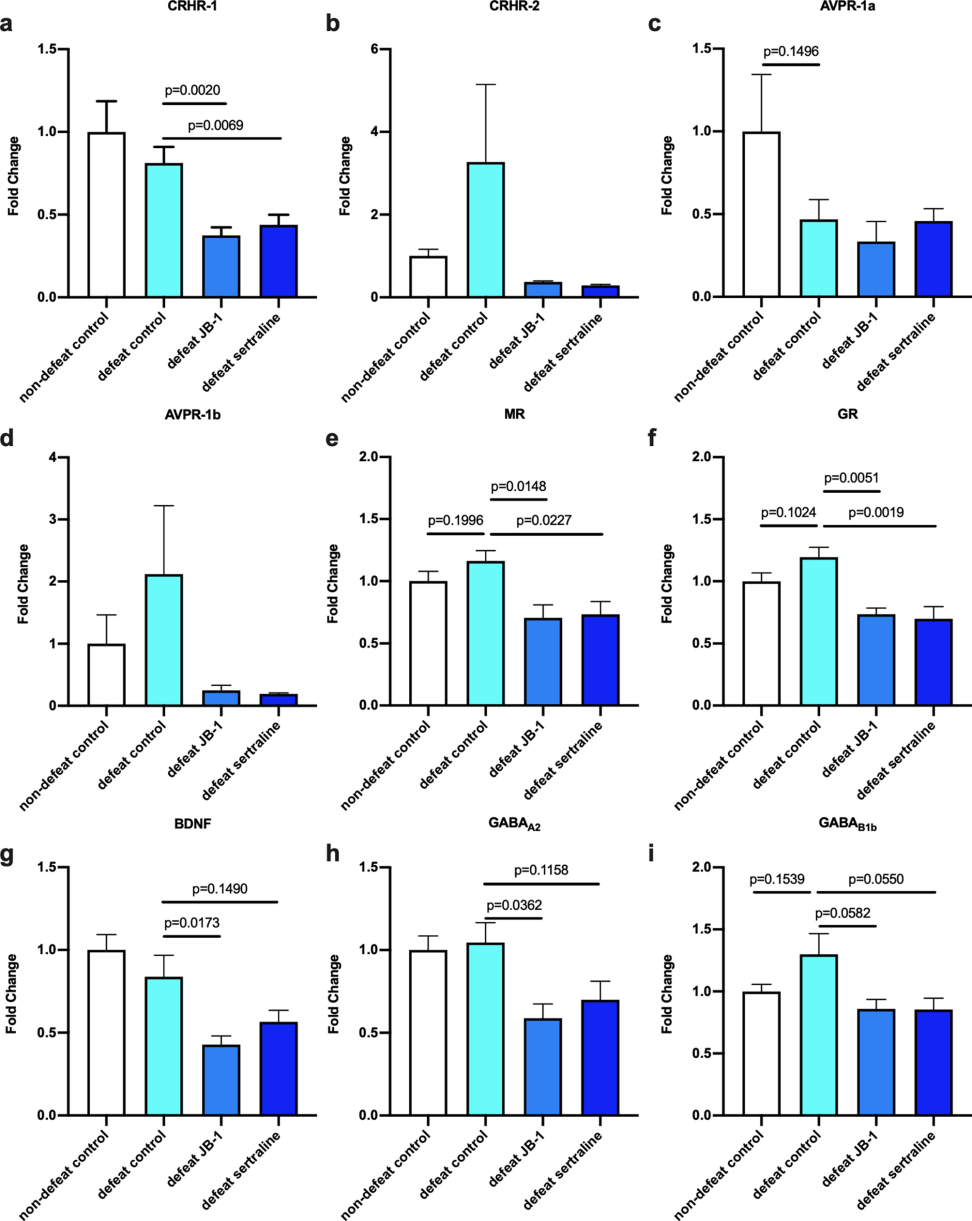
Stress-related gene expression in the hippocampus, represented as fold change compared to mean value of the non-defeat control group (n = 6). (**a**) Corticotropin-releasing hormone receptor-1 (CRHR-1). (**b**) Corticotropin-releasing hormone receptor-2 (CRHR-2). (**c**) Arginine vasopressin receptor-1a (AVPR-1a). (**d**) Arginine vasopressin receptor-1b (AVPR-1b). (**e**) Mineralocorticoid receptor (MR). (**f**) Glucocorticoid receptor (GR). (**g**) Brain-derived neurotrophic factor (BDNF). (**h**) Gamma-aminobutyric acid receptor subunit alpha-2 (GABA_A2_). (**i**) Gamma-aminobutyric acid receptor subunit beta-1b (GABA_B1b_).

We assessed gene expression in the hippocampus of mice 32 days following CSD, with and without post-defeat JB-1 and sertraline treatment; targeting genes related to stress response, social behavior and memory that had been previously identified as being modulated by JB-1 in other models^17,20,21^. For corticotropin-releasing hormone receptor subtypes (CRHR-1 and -2) and arginine vasopressin (AVPR-1a and -1b) in the hippocampus, we found that mice treated with JB-1 or sertraline had significantly lower expression of CRHR-1 mRNA relative to mice in defeat control group (F (2, 15) = 1.391, η^2^*p* = 0.16, defeat control: 0.81 ± 0.097, JB-1: 0.37 ± 0.049, g = 2.32, *p* = 0.0020; sertraline: 0.44 ± 0.061, g = 1.88, *p* = 0.0069) (Fig. 4a). There was no significant difference in CRHR-2 or AVPR-1a and -1b gene expression (Fig. 4b–d).

Defeated mice treated with JB-1 and sertraline also had reduced expression of both mineralocorticoid and glucocorticoid receptors (MR and GR) compared to defeat control (MR: F (2, 15) = 0.098, η^2^*p* = 0.01, defeat control: 1.16 ± 0.084, JB-1: 0.70 ± 0.11, g = 1.93, *p* = 0.0148; sertraline: 0.73 ± 0.10, g = 1.84, *p* = 0.0227) (Fig. 4e) (GR: F (2, 14) = 1.198, η^2^*p* = 0.15, defeat control: 1.20 ± 0.080, JB-1: 0.73 ± 0.051, g = 2.96, *p* = 0.0051; sertraline: 0.70 ± 0.10, g = 2.23, *p* = 0.0019) (Fig. 4f).

There was no significant change in relative BDNF expression in control defeated compared to non-defeat mice 32 days after defeat. However, defeated mice treated with JB-1 had significantly lower BDNF expression (F (2, 15) = 1.403, η^2^*p* = 0.16, defeat control: 0.84 ± 0.13, JB-1: 0.43 ± 0.053, g = 1.69, *p* = 0.0173) (Fig. 4g). For gamma-aminobutyric acid receptor subunit A2 (GABA_A2_) and subunit B1b (GABA_B1b_), we observed significantly lower GABA_A2_ expression in JB-1 treated animals compared to defeat controls, (F (2, 14) = 0.407, η^2^*p* = 0.05, defeat control: 1.05 ± 0.12, JB-1: 0.59 ± 0.086, g = 1.80, *p* = 0.0362) (Fig. 4h) while no differences were observed between groups in the expression of GABA_B1b_ (Fig. 4i).

Treatment of non-defeated mice with JB-1 or sertraline did not result in any significant changes in expression of the genes assessed (see Supplementary Fig. S3).

### JB-1 and sertraline treatment reduced Ly6C^hi^ monocytes and dendritic cell populations, following chronic social defeat

**Figure 5:**
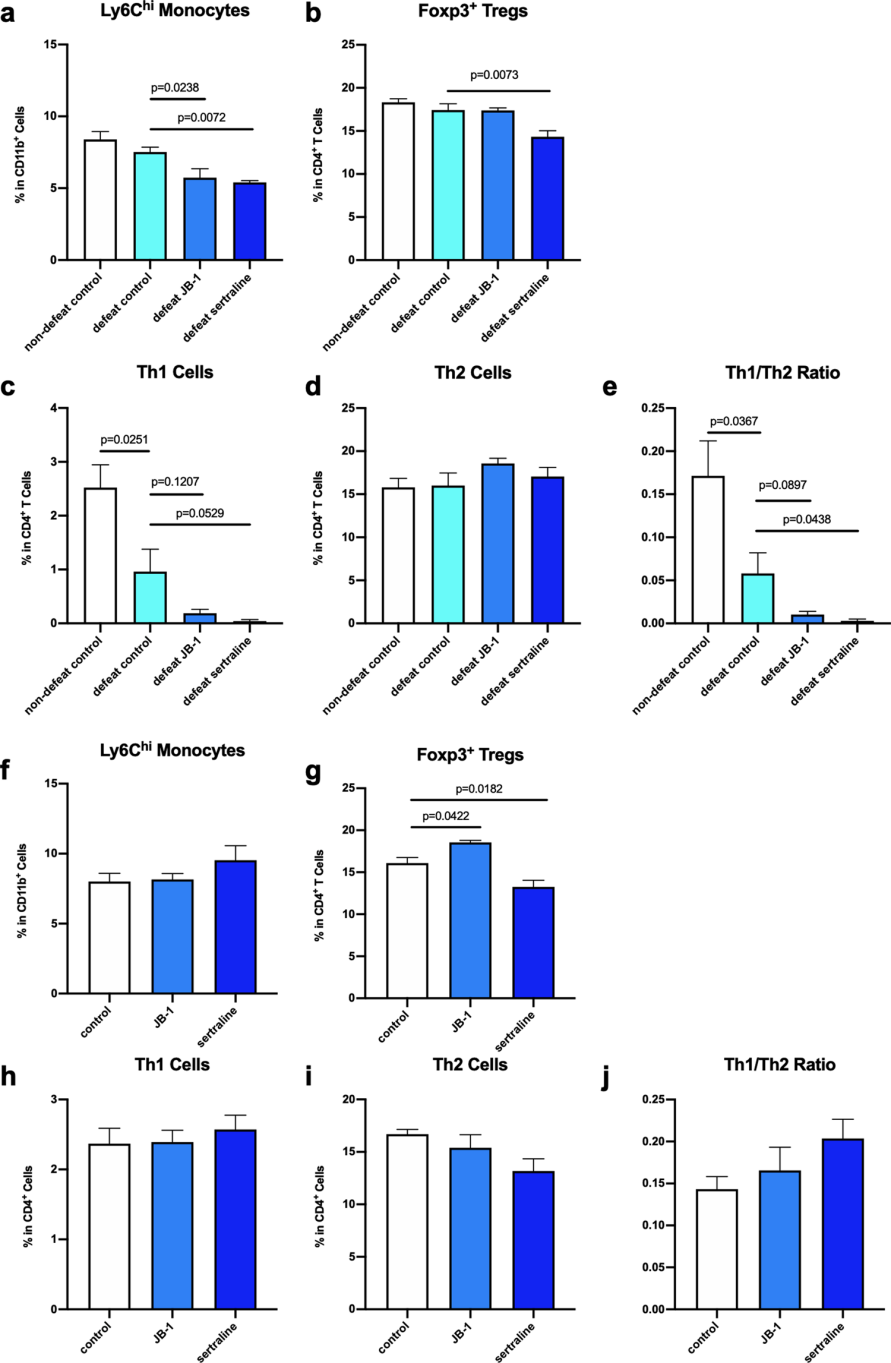
Flowcytometry analysis of splenocytes from the CSD exposed (**a**–**e**) and non-defeated (**f**–**j**) mice following treatment with JB-1 or sertraline (n = 8), showing Ly6C^hi^ monocytes (**a**,**f**), Foxp3^+^ Tregs. (**b**,**g**), Th1 cells (IFN-γ^+^IL-4^−^). (**c**,**h**), Th2 cells (IFN-γ^−^IL-4^+^) (**d**,**i**), and Th1/Th2 ratio (percentage of Th1/percentage of Th2) (**e**,**j**).

Activated monocytes have been recognized as a key inflammatory signal following exposure to social defeat, and increases in Ly6C^hi^ monocytes have been demonstrated to drive anxiety-like behavior following stress exposure^22^. In our study, we found that 32 days after CSD, the population of CD11b^+^Ly6C^hi^ cells was not significantly different between non-defeat and defeat control groups (non-defeat control: 8.39 ± 0.55%, defeat control: 7.52 ± 0.33%, g = 0.79, *p* = 0.2025). However, both sertraline and JB-1 treatment groups had a significantly reduced population of Ly6C^hi^ monocytes (F (2, 15) = 3.696, η^2^*p* = 0.33, JB-1: 5.73 ± 0.62%, g = 1.46, *p* = 0.0238; sertraline: 5.39 ± 0.13%, g = 3.49, *p* = 0.0072) (Fig. 5a). An increase in T regulatory cells (Treg), has been associated with the anxiolytic and antidepressant-like effects of JB-1^23^. Treg were assessed based on expression of Foxp3 in CD4^+^ T cells. The sertraline treatment group had a significantly smaller population of Foxp3^+^ Treg when compared to defeated control (F (2, 15) = 1.108, η^2^*p* = 0.13, defeat control: 8.58 ± 0.34%, sertraline: 6.78 ± 0.39%, g = 1.78, *p* = 0.0039) (Fig. 5b). There was no significant difference in activated dendritic cells (CD80/CD86) among groups (Supplementary Fig. S4a and S4b).

As a measure of general immune balance, stimulated splenocytes were used to assess Th1 and Th2 cell populations based on IFN-γ and IL-4 expression respectively. Defeat alone led to a significant decrease in IFN-γ expressing CD4^+^ (Th1) cells with no significant effect of treatments on this decrease (*t* test: non-defeat control: 2.52 ± 0.42%, defeat control: 0.96 ± 0.42%, g = 1.52, *p* = 0.0251; one-way ANOVA: F (2, 15) = 5.140, η^2^*p* = 0.41, JB-1: 0.19 ± 0.072%, g = 1.06, *p* = 0.1207; sertraline: 0.043 ± 0.029%, g = 1.28, *p* = 0.0529) (Fig. 5c). There was no significant difference in the Th2 (CD4^+^IL-4^+^) cell among groups (Fig. 5d). However, the Th1/Th2 ratio, as an indicator of immune balance, showed a significant decrease after defeat (non-defeat control: 0.17 ± 0.041%, defeat control: 0.058 ± 0.024%, g = 1.39, *p* = 0.0367), and sertraline, but not JB-1, treatment was associated with a significant reduction compared to defeat control (F (2, 15) = 5.367, η^2^*p* = 0.42, JB-1: 0.0103 ± 0.0039, g = 1.13, *p* = 0.0897; sertraline: 0.0031 ± 0.0021%, g = 1.32, *p* = 0.0438) (Fig. 5e).

There was no effect of either JB-1 or sertraline treatment on Ly6C^hi^ monocytes in non-defeated mice (Fig. 5f). However, JB-1 increased, and sertraline decreased, the population of Foxp3^+^ T cells compared the control group (F (2, 15) = 2.404, η^2^*p* = 0.24, control: 16.08 ± 0.68, JB-1: 18.55 ± 0.25, g = 1.96, *p* = 0.0422; sertraline: 13.25 ± 0.81, g = 1.54, *p* = 0.0182) (Fig. 5g). JB-1 or sertraline treatment alone had no effect on Th1 and Th2 cells (Fig. 5h–j) or CD80^+^ and CD86^+^ dendritic cells (Supplementary Fig. S4c and S4d).

### Serum CCL-2 was decreased after JB-1/sertraline treatment on mice after chronic social defeat

**Figure 6:**
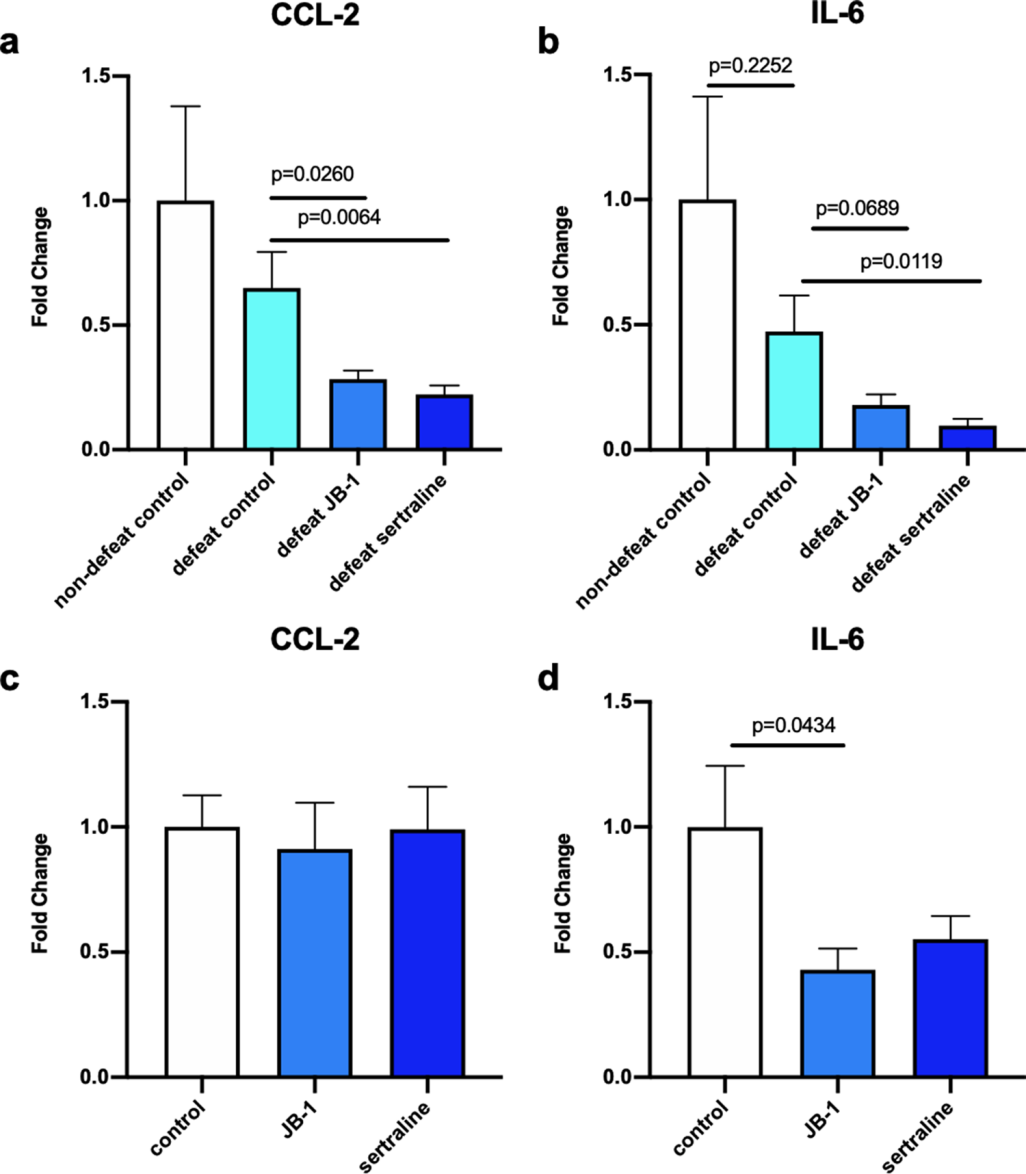
Serum Chemokine (C–C motif) ligand-2 (CCL-2) an Interleukin-6 (IL-6) levels following sertraline or JB-1 treatment in (**a**,**b**) CSD exposed (n = 10–12) and (**c**,**d**) non-defeated (n = 8) mice. Data is expressed as fold change compared to corresponding non-defeated control.

In assessing serum levels of 18 cytokines/chemokines in mice following CSD, we found that relative levels of CCL-2, a major chemoattractant for activated monocytes, were significantly decreased in both JB-1 and sertraline treatment groups compared to defeat control. (F (2, 29) = 4.390, η^2^*p* = 0.23, defeat control: 0.649 ± 0.145, JB-1: 0.284 ± 0.034, g = 1.02, *p* = 0.0260; sertraline: 0.222 ± 0.036, g = 1.22, *p* = 0.0064) (Fig. 6a). In addition, sertraline treatment led to a significant reduction in IL-6 compared to defeated control (F (2, 28) = 5.237, η^2^*p* = 0.27, defeat control: 2.552 ± 0.775, sertraline: 0.523 ± 0.147, g = 1.18, *p* = 0.0119) (Fig. 6b). The results of additional cytokines/chemokines analysed are shown in Supplementary Table S2.

In the non-defeated cohorts, we did not find significant difference among groups in CCL-2 (Fig. 6c). However, we did find JB-1 treatment alone led to significant reduction of IL-6 (F (2, 20) = 2.629, η^2^*p* = 0.21, control: 1.000 ± 0.2440, JB-1: 0.4295 ± 0.0854, g = 1.21, *p* = 0.0434) (Fig. 6d). No significant difference was found in the other cytokines and chemokines assessed (see Supplementary Table S3).

## Discussion

Here we report that treatment with JB-1 or sertraline, initiated 48 h following chronic social defeat, led to a greater persistence of aggressor avoidance and deficits in social behavior compared to control defeated mice. Treatment with JB-1, post-defeat, had no effect on anxiety-like behaviour. The findings contrast those of our previous study which demonstrated that JB-1 treatment concurrent with CSD results in attenuation of both social behavior deficits and anxiety-like behaviors^15^. Thus, it appears that, depending on the temporal relationship between stress exposure and treatment with the bacteria, microbes identified as having anxiolytic/anti-depressant like effects have the potential to potentiate detrimental effects of traumatic events and/or stress.

For mice in the defeat control group, the reduction in social interaction that occurs immediately following stress exposure was not observed 4 weeks later. In the same 4 week period, aggressor avoidance behavior was lost in approximately 50% of these mice, suggesting some mice do not develop long-term fear memory in this CSD model^15^. In a previous study we identified that anxiety associated behaviours which occur immediately following CSD are not apparent 3 weeks later but did not observe a loss in aggressor avoidance behaviour^15^. This difference may be due to the additional 10 days of recovery time in the current protocol. However, treatment with either JB-1 or sertraline resulted in persistence of attenuated social interactions and avoidance behaviour in all the mice tested. That this is a persistence of the effects of CSD rather than actions of the treatment alone is supported by the observation that neither JB-1 nor sertraline altered these behaviours in non-defeated mice. We also noted that in non-defeated mice, regardless of treatment, there was a reduced interaction ratio between the first and second aggressor avoidance test. This likely reflects that in non-defeated mice the initial exposure is one of social novelty, which is lessened in the second exposure.

Using a similar CSD model, Ishikawa et al. found a comparable decrease in percentage time spent in aggressor avoidance zone between 1 day and 4 weeks following stress exposure^24^. However, Hammamieh et al. demonstrated that fear memory persisted 4 weeks after repeated exposure to conspecific trained aggressors^25^. The difference between persistence of behaviours may be due to the distinct models used (chronic social defeat, 5 min daily for 10 days by retired-breeder CD1 mice, vs cage-within-cage intruder exposure, 6 h daily for 5–10 days by 6-week old SJH albino mice).

There have been few previous studies addressing the use of microbe-based treatments following stress exposure. Hassell et al.^14^ reported that rats immunized with heat killed *M. vaccae*, 1 day following fear conditioning, did not show altered fear expression but did have improved fear extinction. It is difficult to compare the work of Hassell et al. with our current study as we used a different model (fear conditioning vs CSD), different species (rat vs mouse) and studied natural loss of the fear response rather than active extinction. Furthermore, it is possible there are distinct mechanisms of action and effects on brain when comparing subcutaneous administration of *M. vaccae* with oral treatment of JB-1.

With regard to sertraline effects, our observations may be in keeping with previous studies indicating that SSRI treatment can modulate fear conditioning^26,27^. Montezinho et al. demonstrated that escitalopram differentially affected distinct stages of contextual fear conditioning. Escitalopram significantly decreased the conditioned responses when administered 30 min before the recall test. However, when applied immediately after acquisition, during consolidation, it enhanced freezing time during fear recall, indicating that escitalopram potentiates memory consolidation^26^. Moreover, serotonin transporter knockout has been correlated with retention of contextual fear memory^28^. Such effects are thought to be due to an important role for the serotonergic system in learning and memory processes, particularly during the encoding and consolidation phases^29^. The time window immediately following fear conditioning or trauma exposure seems to be critical to the fear enhancing effects of SSRIs as Wang et al. demonstrated that administration of paroxetine after a space of 1 week following conditioned fear stress (electric shock) combined with single-prolonged stress (immobilization) reduced anxiety-like behaviour and fear conditioned freezing in mice^30^. There is evidence that environmental context is critical to the action of SSRIs. Alboni et al.^31^ demonstrated that previously stressed mice treated with fluoxetine in an enriched environment improved their depression-like phenotype whereas those treated in a stressful condition demonstrated more marked depressive behaviours than controls. Such findings support the “undirected susceptibility to change’ hypothesis, which posits that SSRI treatment does not drive changes in mood per se but increases brain plasticity allowing for change driven by the quality of the environment^31,32^. Such increased plasticity in a period soon after defeat may help in the persistence of aggressor related memories and suggests future studies should examine neurogenesis in response to sertraline and JB-1 following CSD.

The effects of JB-1 and sertraline following CSD may also reflect the clinical picture in relation to early-stage pharmacological prophylaxis for PTSD. Such approaches have had, at best, mixed results. Early findings by Pitman et al.^33^ on propranolol (a beta-adrenergic blocker) found that patients treated with propranolol had reduced negative responses during script-driven imagery of the traumatic event than placebo, although overall there was no significant difference in the number of subjects that went on to develop PTSD. However, Shalev et al.^34^ found that early intervention in trauma exposed subjects with the SSRI escitalopram, led to a slightly higher rate in PTSD prevalence while a more recent clinical trial found that early administration of escitalopram had no effect on PTSD development in individuals exposed to trauma^35^.

In the current study, we limited our investigation of the brain to gene expression in the hippocampus. Hippocampal changes after exposure to traumatic events have been correlated with chronic psychiatric symptoms^36–38^. The expression of the CRHR-1 and -2, and AVPR-1a and -1b in the hippocampus have been demonstrated to be related to HPA axis activation and stress induced depressive and anxiety-like behavior^39,40^. Moreover, changes in CRHR-1 expression have been linked with fear conditioned memory and cognitive deficits^41,42^. Thus, our observation that defeated mice treated with either JB-1 or sertraline had reduced CRHR-1 in the hippocampus may be functionally related to the behavioral difference in aggressor-approach avoidance test, as an indicator of fear conditioned memory.

Exposure to CSD followed by treatment with either JB-1 or sertraline, resulted in reduced expression of hippocampal MR and GR. Both receptor types have been implicated in the consolidation of fear memory and impaired fear extinction. MR and GR in hippocampus regulate the hypothalamic pituitary adrenal axis (HPA axis) and are thus involved in the modulation of stress response, including fear, memory and anxiety^43–45^. Lower MR functionality can result in an increased susceptibility for negative stress responses^46^. Brinks et al.^47^ found that forebrain MR gene inactivated mice had higher arousal and less locomotor activity, as well as more freezing in cue-related fear behavior test. In relation to GR, the ameliorating effects of cannabinoid receptor agonist on contextual fear extinction are prevented by blocking GR in the hippocampus, indicating that GR activation is positively correlated with fear extinction^48^.

Changes in hippocampal BDNF expression may also play an important role in mediating the observed effects of JB-1 on behaviour. Our data suggest that post-CSD administration of JB-1 leads to reduced BDNF expression in the hippocampus. BDNF has been closely linked to stress responses and memory of fear experience, with stronger fear memory correlated with lower levels of BDNF in hippocampus^49^ while intrahippocampal administration of BDNF induces extinction of conditioned fear even in the absence of extinction training^50^. It is possible that reduced levels of BDNF in the hippocampus in turn inhibit or retard loss of fear related memories, maintaining aggressor avoidance and social behavior deficits.

Overall, our results indicate that both JB-1 and sertraline lead to alterations in the expression of several molecular factors involved in fear related memory and stress responses in the hippocampus of defeated mice. Such observations warrant a more detailed investigation of a potential causal relationship between the observed gene expression changes and the prolongation of CSD induced alterations in behavior.

It is established that psychological stress can lead to immunological alterations and vice versa^51,52^. While an increase in markers of inflammation has been associated with both CSD in mice^15^ and PTSD in humans^53^^,^ the causal relation between the immune system and associated behavioral changes is not well understood. The observed effects of JB-1 and sertraline on the immune system of defeated mice could generally be described as anti-inflammatory, with a decrease in Th1 cells and activated monocytes. A possible explanation of these changes could be a compensatory activation of the HPA axis following the reduction of GR/MR expression. Harris et al. found that low level of GR and MR could not only lead to retention of fear memory, but also impaired negative feedback of HPA axis, which was correlated with higher level of corticosteroid in plasma^54^. HPA axis hyperactivation is linked with to a series of immunosuppressive effects including lower IFN-γ and IL-6 levels^55,56^. Further studies on hyperactivity of HPA axis could be conducted to explore the possible mechanism of compensatory hyperactivity of HPA axis after GR/MR reduction.

Monocytes are one of the major cellular components of an inflammatory response. In our study, we found that defeated mice treated with JB-1 or sertraline had fewer Ly6C^hi^ monocytes, with associated lower serum levels of CCL-2, one of the major chemoattractants for monocytes. A potential causal relationship between a decrease in Ly6C^hi^ monocytes and persistence of aggressor avoidance and reduction in social preference is unclear. However, it has been demonstrated that circulating Ly6C^hi^ monocytes can migrate to the brain^57^ and once in the brain these cells can influence hippocampal neurogenesis and cognition^58^. Another possibility is that the change in monocytes activation is instead related to the anxiolytic effects of sertraline and JB-1. Anxiety-like behavior induced by chronic social stress was demonstrated to be linked with higher numbers circulating activated monocytes (CD11b^+^Ly6C^hi^ cells) and greater macrophage trafficking to the brain^22^. Furthermore an increase in circulating activated monocytes has been associated with stress resilience in mice^59^. While no anxiolytic effects of JB-1 were observed in the current study, possibly because of the limited anxiety like behaviour exhibited by defeated mice at the time-points studied, previous studies have indicated the ability of JB-1 to reduce both trait^17^ and state^15^ anxiety-like behavior in mice. The relationship between inflammation and cognition is clearly complex and investigation of the potential effects of immunomodulation on event related fear memory is warranted.

In conclusion, this study demonstrates, for the first time, that an organism previously identified as having both anxiolytic and antidepressant activities can, under certain circumstances perpetuate, rather than prevent, behavioral deficits associated with stress exposure. In addition, we demonstrate that treatment with the SSRI, sertraline, in the immediate aftermath of a chronic stressor, likely corresponding to the memory consolidation phase, can also exacerbate negative sequelae. Under both treatment conditions, such effects are associated with changes in hippocampal expression of genes associated with fear memory and modulation of the immune system, most markedly a decreased in circulating inflammatory monocytes. This data invites further exploration of the relationship between bacteria in gut and trauma or stressor related memory, particularly during the consolidation phase. The similarity of effects between JB-1 and sertraline also encourages studies focusing on possible shared mechanistic pathways between the probiotic and SSRI. In this regard, there is evidence that both JB-1 and oral SSRIs activate the vagus nerve^17,60–63^, and subdiaphragmatic vagotomy prevents certain effects of both treatments on the brain and behaviour of mice^17,60,63^.

Given that this study identifies the potential for detrimental effects of both JB-1 and sertraline in stress/trauma related conditions, future work examining the potential ramifications of early intervention following trauma on patients at risk for development of PTSD are necessary.

## Methods

### Animals

Male C57BL/6 mice (6–8 weeks old) and CD-1 (retired breeders) were acquired from Charles River (Montreal, Canada). All mice were acclimated for 1 week before the instigation of experiments, in standard conditions (12-h light–dark cycle, 5 a.m.–5 p.m.). All mice were fed ad libitum with standard chow and water. The CSD experiment was conducted in 4 cohorts (3–4 mice per group per cohort), and the non-defeat experiment was conducted in 2 cohorts (4 mice per group per cohort). All mice were handled for 3 days before 10-day CSD or control treatment, and the intervention and behavior tests were conducted by same researcher. All experiments followed Canadian Council on Animal Care guidelines and were approved by the McMaster Animal Research Ethics Board.

### Chronic social defeat

CD-1 aggressors were screened and chronic social defeat was conducted according to a standard protocol described by Golden et al^19^. Briefly, CD-1 aggressors were housed singly in rat cages (with a clear perforated Plexiglas divider in the middle and normal bedding) 24 h before the first session of defeat for acclimation. On the first test day, a C57BL/6 intruder was placed into the CD1 aggressor compartment to allow 5 min of contact with the aggressor and then moved to the opposite compartment, with olfactory, visual and auditory contacts allowed. This defeat procedure was repeated for 10 days, with the intruder placed into a novel aggressor cage daily. For the C57BL/6 mice in all non-defeated groups, two C57BL/6 mice were housed in the same cage separated by a divider as described above, and the partner of each was changed daily. After the final session was completed, all intruders were housed singly. Only the susceptible mice (with an interaction ratio < 1 according to the aggressor-approach avoidance screening carried out 1 day after the final session of defeat) were used for further treatment and tests.

### JB-1 and sertraline administration

48 h following the final defeat session, mice in the JB-1 treated group were administered 1 × 10^9^ CFU of *Lactobacillus rhamnosus* JB-1 (a gift from Alimentary Health Ltd., Cork, Ireland) in 200 μl of phosphate-buffered saline (PBS) by oral gavage as previously described^15^. Non-JB-1 treated animals received an equivalent volume of PBS via gavage, including those in the SSRI-treated groups. SSRI treated group received sertraline (6 mg/kg, dissolved in tap water, MilliporeSigma Canada Co., Oakville, Canada), via a 50 ml conical centrifuge tube with fitted sipper, while all animals not in the sertraline treatment group received tap water via the same method. We have previously demonstrated that this dose of sertraline and treatment protocol has antidepressant-like effects in mice^60^. All treatments were provided daily until the end of the experiment.

### Behavioral tests

Behavioral tests were conducted as previously described^15,17^. All intruders were screened for aggressor avoidance behaviour 1 day after the final session of social defeat and then randomly assigned to their treatment groups. Following 18 days of treatment, the mice were tested in the open field test (OFT), light–dark test (LDT), elevated plus maze (EPM), 3-chamber sociability test and aggressor avoidance test. Interaction ratio was applied as result in aggressor avoidance test, calculated by time spent in aggressor area/time spent in empty area. The novel mouse used in sociability test was same sex, strain and age as the test mouse. The “aggressor” in the aggressor avoidance test was a CD1 mouse, which passed the aggressor screening test but was not utilised in the CSD paradigm. A 4-day interval was inserted between each test in order to reduce the impact of previous tests on behavior. All behavioral tests were conducted during the dark phase under normal light conditions and with a 1 h period of habituation in the testing room. OFT, LDT and EPM results were recorded by Motor Monitor software (Kinder Scientific, Poway, Canada). The results of 3-chamber sociability test and aggressor approach-avoidance test were recorded by EthoVision XT software (Noldus, Leesburg, Canada). The apparatus was thoroughly cleaned with distilled water and dried between animals tested.

### Flowcytometry

Mice were euthanized 2 days following the final behavioral test using small animal guillotine. A single cell suspension of splenocytes was made after tissue harvest and lysis of red blood cells. Splenocytes (1 × 10^6^) were stained for regulatory T cells (CD3-APC-eFluor780, CD4-FITC, CD25-APC, Foxp3-PE), monocytes (CD11b-PE, Ly6C-APC, CCR2-FITC, CX3CR1-PerCP/Cy5.5) and dendritic cells (CD11c-APCeFluor780, CD80-PerCP, CD86-APC, MHC II-FITC). Splenocytes stimulated by phorbol 12-myristate 13-acetate (PMA) and ionomycin, were stained for markers of Th1 and Th2 cells (IFN-γ-APC, IL-4-PerCP-eFluor710). Cells were stained by extracellular antibodies at first, then cytofix was applied and followed by staining with intracellular antibodies. All conjugated antibodies were from Invitrogen, ThermoFisher Scientific, USA, and diluted by 1:200 before adding to the plates. The flowcytometry results were analysed by FlowJo software (FlowJo LLC, USA), with doublets and cell debris excluded by FSC and SSC gating.

### Cytokine/chemokine analysis

Serum was collected from trunk blood directly after euthanization from common carotid artery. The levels of interleukin (IL)-1a, -1b, -2, -4, -5, -6, -10, -12, -13, -17A, chemokine (C-X-C motif) ligand-1, -2, -5, C–C motif ligand-2 (CCL-2), tumor necrosis factor-alpha (TNF-α), and interferon-gamma (IFN-γ) were determined through Multiplex Cytokine/Chemokine Analysis (Eve Technologies, Calgary, Canada). Data is presented as fold changes in the figures, and pg/ml in the Supplementary Tables.

### RT-qPCR

Hippocampus as a whole was collected and RNA was extracted using *mir*Vana miRNA isolation kit (Thermofisher Scientific, USA) at the day of euthanization by dissection. The quality of the extracted RNA was analysed using NanoDrop Spectrophotometer ND-1000 (Thermofisher Scientific, USA) and DNA contaminants removed using Invitrogen TURBO DNA-*free* kit. cDNA was created using the Applied Biosystems High Capacity cDNA Reverse Transcription kit (Thermofisher Scientific, USA).

Primers were chosen based on previous studies^17,20,64^ and all primer sequences are listed in Supplementary Table S1. PowerUP SYBR Green Master Mix (Applied Biosystems, Life Technologies, USA) containing ROX Passive Reference Dye was mixed with cDNA and the appropriate primers. The qPCR reaction was carried out in fast mode (uracil-DNA polymerase activation 50 °C, 2 min; Dual-Lock DNA polymerase 95 °C, 2 s; denaturation 95 °C, 1 s; annealing/extension 60 °C, 30 s; number of cycles: 40) using QuantStudio3 (Applied Biosystems). The transcripts were normalized to the housekeeping gene glyceraldehyde-3-phosphate dehydrogenase (GAPDH) and quantified using the ΔΔCt method, with related fold change expressed as 2^(−ΔΔCt)^. Each sample was run as a triplicate.

### Statistical analysis

Data were analysed by the software GraphPad Prism 8.0 by *t* test (for comparisons between non-defeat control and defeat control groups) and one-way ANOVA with Bonferroni-corrected post hoc tests (for comparison between treatment groups and appropriate vehicle control). In figures results are illustrated as mean ± standard error of the mean (SEM) unless otherwise stated. *p* value smaller than 0.05 was considered as statistically significant. Effect size was analysed as partial eta squared (η^2^p) for main effects, and Hedge’s G (g) was applied for post-hoc analysis.

## Supplementary information

Supplementary Information.
