# Publisher Correction: Increased persistence of avoidance behaviour and social deficits with *L.rhamnosus* JB-1 or selective serotonin reuptake inhibitor treatment following social defeat

**DOI:** 10.1038/s41598-020-75605-5

**Published:** 2020-10-23

**Authors:** Yunpeng Liu, Kailey Steinhausen, Aadil Bharwani, M. Firoz Mian, Karen‑Anne McVey Neufeld, Paul Forsythe

**Affiliations:** 1 McMaster Brain-Body Institute, The Research Institute of St. Joseph’s Hamilton, Hamilton, Canada; 2 Department of Pathology and Molecular Medicine, McMaster University, Hamilton, Canada; 3 Michael G. DeGroote School of Medicine, McMaster University, Hamilton, Canada; 4 Department of Medicine, McMaster University, Hamilton, Canada; 5 Firestone Institute for Respiratory Health, St. Joseph’s Healthcare and Department of Medicine, McMaster University, Hamilton, Canada

Correction to: *Scientific Reports* 10.1038/s41598-020-69968-y, published online 10 August 2020

In the original version of this Article, the author Karen-Anne McVey Neufeld was incorrectly indexed. This error has now been corrected.

