# Supplementary material for: Increased persistence of avoidance behaviour and social deficits with L.rhamnosus JB-1 or selective serotonin reuptake inhibitor treatment following social defeat
Source: Sci Rep. 2020 Aug 10;10:13485. doi: 10.1038/s41598-020-69968-y (PMC7417579; doi:10.1038/s41598-020-69968-y)
Supplement: Supplementary file 1 — Supplementary Information. [file 41598_2020_69968_MOESM1_ESM.docx]

**Supplementary Information**

**Increased persistence of avoidance behaviour and social deficits with *L.rhamnosus* JB-1 or selective serotonin reuptake inhibitor treatment following social defeat.**

Yunpeng Liu^1^, Kailey Steinhausen^1^, Aadil Bharwani^1,2,3^, M. Firoz Mian^1^, Karen-Anne McVey Neufeld^1,2^, Paul Forsythe^1,4,5*^

^1^McMaster Brain-Body Institute, The Research Institute of St. Joseph’s Hamilton, Hamilton, Canada

^2^Department of Pathology & Molecular Medicine, McMaster University,

Hamilton, Canada

^3^Michael G. DeGroote School of Medicine, McMaster University, Hamilton, Canada

^4^Department of Medicine, McMaster University,Hamilton, Canada

^5^Firestone Institute for Respiratory Health, St. Joseph’s Healthcare and Department of Medicine, McMaster University, Hamilton, Canada

*** Corresponding author:** Paul Forsythe, Ph.D. E-mail: forsytp@mcmaster.ca

**Supplementary Table S1.** Primer sequences of brain PCR.

| **Target mRNA** | **Forward (F) and Reverse (R) Primers** |
| --- | --- |
| GAPDH | F: 5’-TGG CCT CCA AGG AGT AAG AAA C-3’  R: 5’-GGG ATA GGG CCT CTC TTG-3’ |
| CRHR1 | F: 5’-TGG TGG CCT TTG TCC TCT TC-3’  R: 5’-AAA GCC GAG ATG AGG TCC C-3’ |
| CRHR2 | F: 5’-TGA CCA GCC CTT TAC CAA GGT-3’  R: 5’-CCG ACT GAA AGC CAG CAT TC-3’ |
| AVPR1a | F: 5’-GGG ATA CCA ATT TCG TTT GG-3’  R: 5’-AAG CCA GTA ACG CCG TGA T-3’ |
| AVPR1b | F: 5’-TCT ACT CTC CGT CTT AGC CTT AAC CT-3’  R: 5’-CTC CAT CCA CCT GCT CCA A-3’ |
| MR | F: 5’-ATG GAA ACC ACA CGG TGA CCT-3’  R: 5’-AGC CTC ATC TCC ACA CAC AAG-3’ |
| GR | F: 5’-AAA GGT GGC GCT TAT GTA CTT AGA G-3’  R: 5’-CGT GCG GAG GCT GCA T-3’ |
| BDNF | F: 5’ CTG ACA CTT TTG AGC ACG TCA TC-3’  R: 5’-CAC CCG GGA AGT GTA CAA GTC-3’ |
| GABA_Aα2_ | F: 5’-CCA AAG ATC CTG TCC TCT CTA CCA-3’  R: 5’-GGC TTC AGC TGG CTT GTT CT-3’ |
| GABA_B1b_ | F: 5’-CGA GGT GAA TGG CAG TCT GA-3’  R: 5’-CAC GGT TTT CCT TCT CCA ACA-3’ |

Abbreviations: GAPDH: glyceraldehyde 3-phosphate dehydrogenase, CRHR: corticotropin releasing hormone receptor, AVPR: arginine vasopressin receptor, MR: mineralocorticoid receptor, GR: glucocorticoid receptor, BDNF: brain-derived neurotrophic factor, GABA_Aα2_: gamma-aminobutyric acid receptor A subunit alpha-2, GABA_B1b_: gamma-aminobutyric acid receptor B subunit 1b.

**Supplementary Table S2.** Serum analysis of cytokines and chemokines in the CSD experiment (mean±SEM, presented as pg/ml, n=12).

|  | **non-defeat control** | **defeat control (p value*)** | **defeat JB-1**  **(p value**)** | **defeat sertraline**  **(p value**)** |
| --- | --- | --- | --- | --- |
| **IL-1a** | 423.4±72.5 | 275.4±64.3  (p=0.1422) | 286.1±35.9  (p>0.9999) | 277.3±39.3  (p>0.9999) |
| **IL-1b** | 3.1±1.1 | 1.2±0.4  (p=0.0961) | 1.0±0.3  (p>0.9999) | 1.2±0.3  (p>0.9999) |
| **IL-2** | 6.6±1.9 | 7.4±1.5  (p=0.7228) | 8.7±1.2  (p>0.9999) | 11.8±1.7  (p=0.1411) |
| **IL-4** | 0.6±0.2 | 0.2±0.1  (p=0.0659) | 0.1±0.0  (p>0.9999) | 0.1±0.0  (p=0.9346) |
| **IL-5** | 9.9±2.2 | 3.5±0.5  (p=0.0115) | 6.4±1.3  (p=0.0523) | 3.4±0.4  (p>0.9999) |
| **IL-6** | 6.0±2.5 | 2.8±0.9  (p=0.2252) | 1.1±0.3  (p=0.0689) | 0.6±0.2  (p=0.0119) |
| **IL-10** | 9.4±4.0 | 1.3±0.3  (p=0.0431) | 2.7±0.7  (p=0.1042) | 1.7±0.3  (p>0.9999) |
| **IL-12** | 17.8±8.2 | 15.6±3.7  (p=0.8035) | 11.4±2.4  (p=0.7671) | 4.9±1.0  (p=0.0155) |
| **IL-13** | 11.8±1.6 | 9.8±0.7  (p=0.2881) | 7.3±0.8  (p=0.0403) | 7.4±0.5  (p=0.0304) |
| **IL-17A** | 2.0±0.6 | 1.7±0.2  (p=0.6272) | 1.7±0.4  (p>0.9999) | 1.4±0.3  (p>0.9999) |
| **CXCL-1** | 165.9±27.3 | 205.9±20.0  (p=0.2498) | 169.4±18.4  (p=0.5869) | 142.2±20.2  (p=0.0824) |
| **CXCL-2** | 75.0±7.7 | 68.0±5.7  (p=0.5039) | 81.4±6.8  (p=0.4594) | 73.9±5.9  (p>0.9999) |
| **CXCL-5** | 4 895.8±798.4 | 4 616.6±545.2  (p=0.7723) | 4 545. 3±473.1  (p>0.9999) | 3 623.9±471.7  (p=0.5197) |
| **CCL-2** | 22.8±8.7 | 14.8±3.3  (p=0.3819) | 6.5±0.8  (p=0.0260) | 5.1±0.8  (p=0.0064) |
| **TNF-α** | 6.8±3.6 | 1.0±0.2  (p=0.1192) | 0.6±0.1  (p=0.2114) | 0.5±0.1  (p=0.1521) |
| **IFN-γ** | 0.7±0.3 | 0.7±0.12  (p=0.9739) | 0.2±0.1  (p=0.1748) | 0.4±0.2  (p=0.4948) |

*: p value calculated by unpaired t test, compared with non-defeat control.

**: p value calculated by one-way ANOVA and post-hoc Bonferroni correction, compared with defeat control.

Abbreviations: IL: interleukin, CXCL: chemokine (C-X-C motif) ligand, CCL-2: chemokine (C-C motif) ligand-2, TNF-α: tumor necrosis factor-alpha, IFN-γ: interferon-gamma.

**Supplementary Table S3.** Serum analysis of cytokines and chemokines in the non-defeat experiment (mean±SEM, presented as pg/ml, n=8).

|  | **non-defeat control** | **JB-1**  **(p value*)** | **sertraline**  **(p value*)** |
| --- | --- | --- | --- |
| **IL-1a** | 614.8±72.2 | 802.9±245.0  (p>0.9999) | 814.6±116.6  (p>0.9999) |
| **IL-1b** | 18.2±10.1 | 6.2±0.6  (p=0.4207) | 6.6±0.9  (p=0.4946) |
| **IL-2** | 47.8±22.8 | 103.3±44.5  (p=0.6452) | 33.6±2.2  (p>0.9999) |
| **IL-4** | 6.5±2.6 | 0.9±0.2  (p=0.0570) | 0.8±0.3  (p=0.0629) |
| **IL-5** | 37.9±11.6 | 13.9±2.2  (p=0.1973) | 31.8±9.6  (p>0.9999) |
| **IL-6** | 50.9±12.4 | 21.9±4.3  (p=0.0434) | 28.1±4.7  (p=0.1447) |
| **IL-10** | 60.8±25.1 | 22.6±1.2  (p=0.1929) | 16.3±2.4  (p=0.1166) |
| **IL-12** | 139.2±69.7 | 46.0±10.2  (p=0.3427) | 35.3±7.3  (p=0.3069) |
| **IL-13** | 17.7±5.6 | 68.23±25.5  (p=0.1092) | 15.8±4.9  (p>0.9999) |
| **IL-17A** | 16.1±4.3 | 10.4±2.0  (p=0.5924) | 9.2±2.2  (p=0.3680) |
| **CXCL-1** | 174.9±15.6 | 207.1±16.9  (p=0.9819) | 173.7±30.1  (p>0.9999) |
| **CXCL-2** | 329.5±37.4 | 317.0±32.8  (p>0.9999) | 363.6±28.7  (p>0.9999) |
| **CXCL-5** | 10,674.3±2,063.4 | 15,931.3±3,177.3  (p=0.3748) | 10,856.1±1,370.2  (p>0.9999) |
| **CCL-2** | 101.6±12.9 | 92.7±18.8  (p>0.9999) | 100.7±17.2  (p>0.9999) |
| **TNF-α** | 24.1±5.3 | 18.5±2.7  (p>0.9999) | 23.2±4.9  (p>0.9999) |
| **IFN-γ** | 2.9±0.8 | 14.6±4.9  (p=0.2279) | 10.2±4.3  (p=0.7344) |

*: p value calculated by one-way ANOVA and post-hoc Bonferroni correction, compared with non-defeat control.

**Fig. S1**

**Time spent in social (mouse) chamber of 3-chamber sociability test, 1 day after 10-day CSD (n=8 per group).**

**
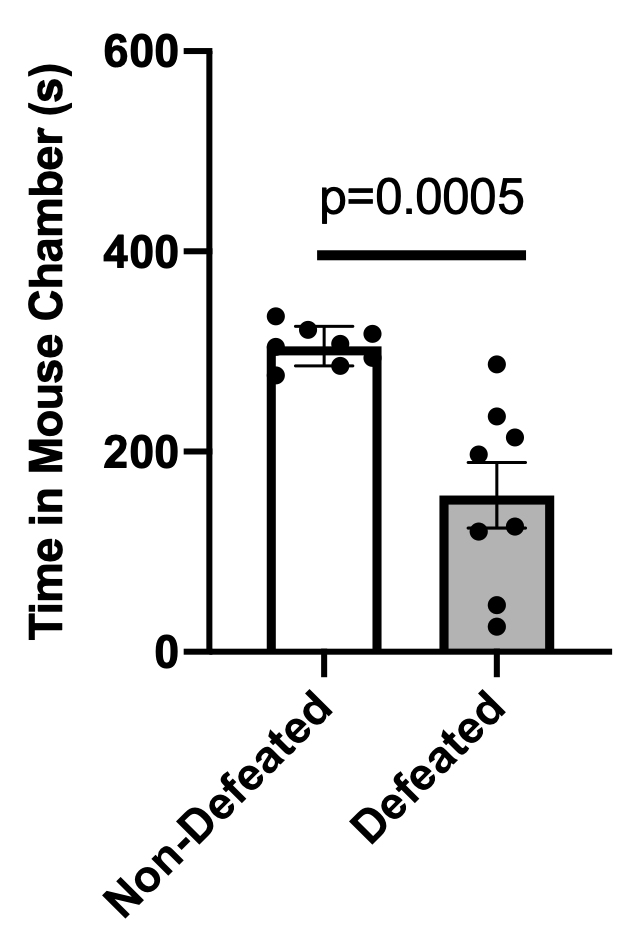
**

**Fig. S2**

**Supplementary behavior data.**

**
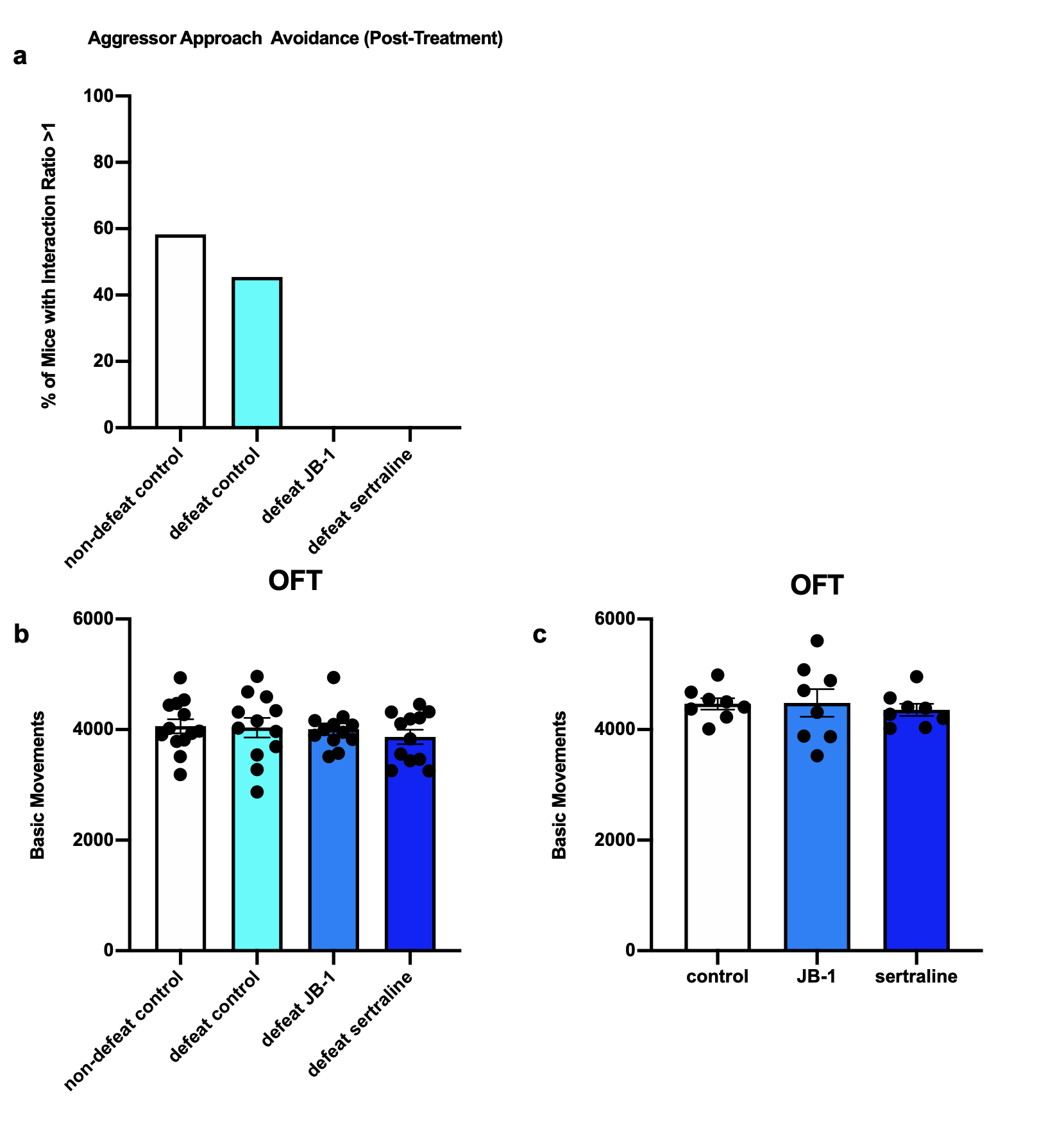
**

**Fig. S3**

**Stress-relate mRNA expression in hippocampus of the non-defeat experiment.**

**
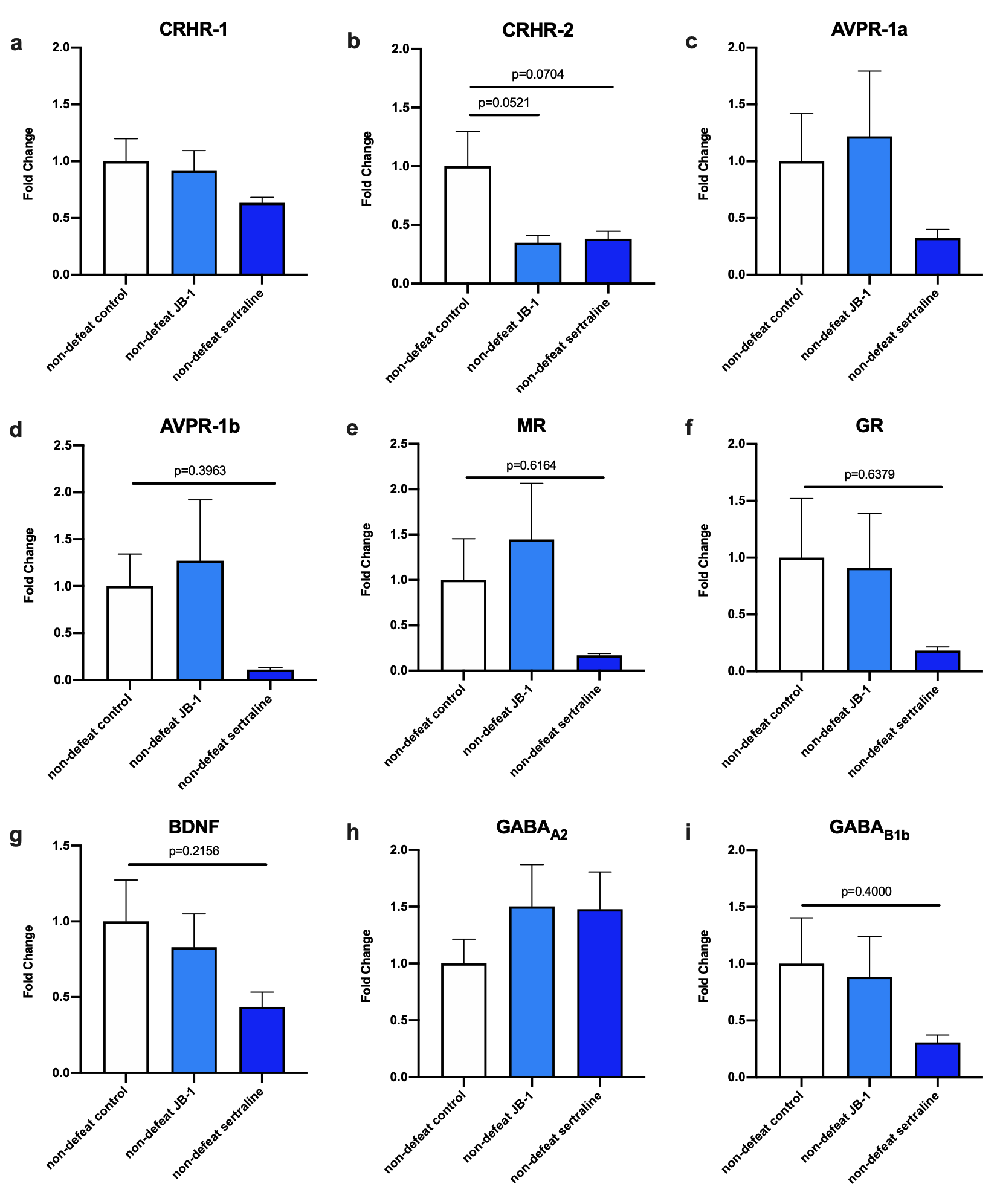
**

**Fig. S4**

**Flowcytometry results of CD80^+^ and CD86^+^ dendritic cells in splenocytes.

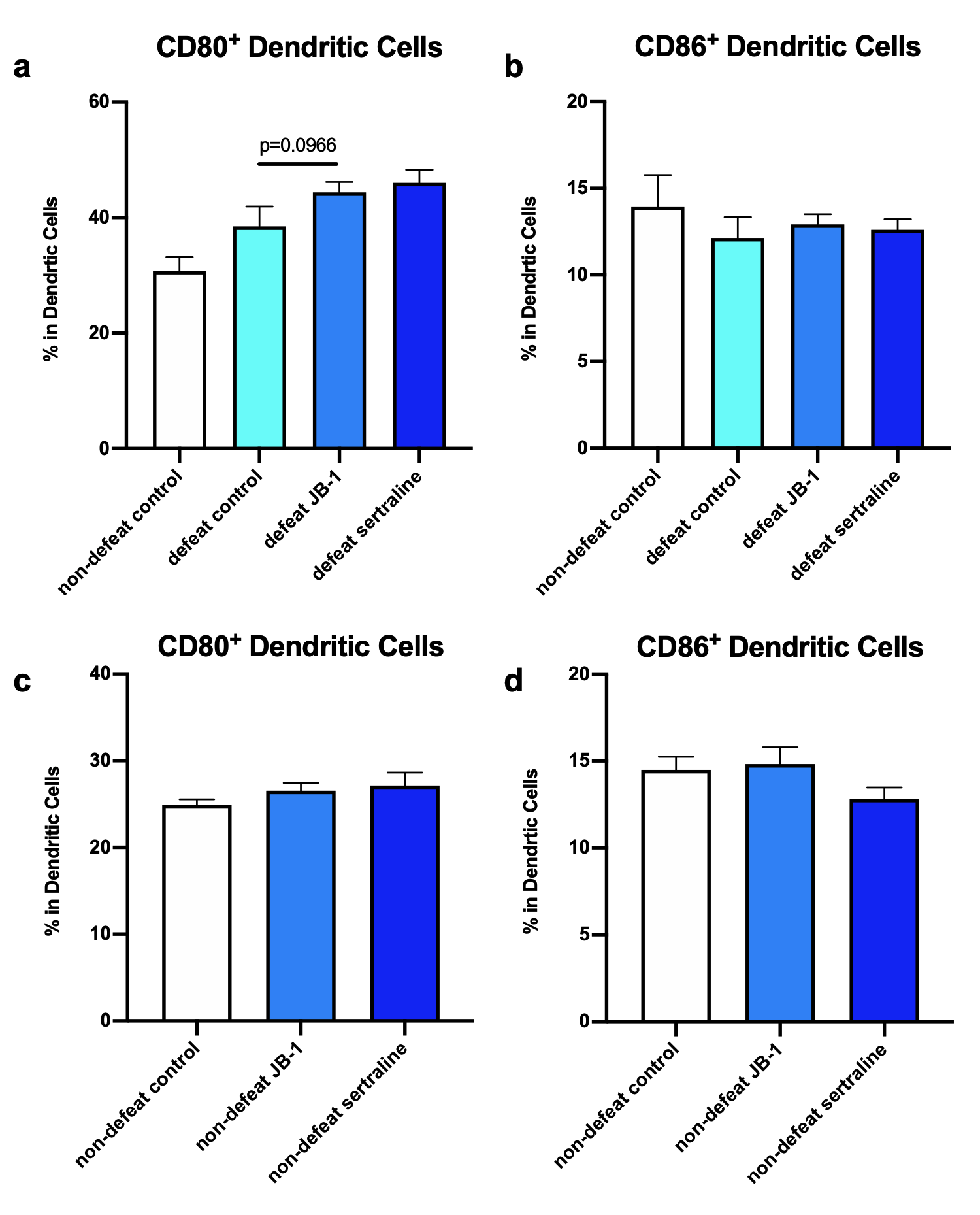
**
